# Draft Genome Sequence of *Ralstonia solanacearum* Race 4 Biovar 4 Strain SD54

**DOI:** 10.1128/genomeA.00890-13

**Published:** 2013-12-19

**Authors:** Weiwei Shan, Xiaohui Yang, Weiqing Ma, Yu Yang, Xiao Guo, Jianhua Guo, Huajun Zheng, Guangcun Li, Bingyan Xie

**Affiliations:** Life Science College, Shandong Normal University, Jinan, Chinaa; Institute of Vegetables and Flowers, Shandong Academy of Agricultural Sciences, Shandong Key Laboratory for Biology of Greenhouse Vegetables, Jinan, Chinab; Plant Protection College, Nanjing Agriculture University, Nanjing, Chinac; Shanghai—MOST Key Laboratory of Health and Disease Genomics, Chinese National Human Genome Center at Shanghai, Shanghai, Chinad; Institute of Vegetables and Flowers, Chinese Academy of Agricultural Sciences, Key Laboratory of Horticultural Crops Biology and Genetic Improvement, Ministry of Agriculture, Beijing, Chinae

## Abstract

*Ralstonia solanacearum* is an important etiological agent that can cause serious bacterial wilt in a very wide range of potential host plants, including ginger. Here, we report the complete genome sequence of *R. solanacearum* SD54, a race 4 biovar 4 (R4B4) strain from a diseased ginger plant in China.

## GENOME ANNOUNCEMENT

*Ralstonia solanacearum* is an aerobic, non-spore-forming, Gram-negative plant pathogenic bacterium and is soil borne and motile with a polar flagellar tuft. It colonizes the xylem, causing bacterial wilt in a very wide range of potential host plants, including ginger. *R. solanacearum* is considered to be a species complex, a heterogeneous group of related but genetically distinct strains (1, 2). Traditionally, the group has been subdivided into 5 races and 4 biovars based on the differences in host range (3, 4) and the acidification of medium during the metabolism of six carbohydrates (maltose, lactose, cellobiose, mannitol, sorbitol, and dulcitol) (5, 6), respectively. An entirely new phylogenetic classification system was proposed recently, consisting of four phylotypes, each further divided into sequevars (1, 7).

One of the major strains that has caused serious bacterial wilt damage in ginger plants in China is *R. solanacearum* race 4 biovar 4 (R4B4) SD54, the draft genome sequence of which is reported here.

The nucleotide sequence was determined using a 454 GS FLX sequencer, and assembly was performed using the GS *de novo* assembler software version 2.3 (8). A total of 280,325 reads, including up to 100,016,604 bp, were obtained, which represents a 17.6-fold coverage of the genome. In addition, a 100-fold coverage of the 3-kb mate-pair library was constructed and sequenced using an Illumina HiSeq 2000. After scaffold construction and gap filling, we finally obtained the *R. solanacearum* SD54 draft genome sequence of 5,648,859 bp distributed in 175 contigs, with a G+C content of 66.9%.

Putative protein coding sequences (CDSs) were predicted using Glimmer (9) and GeneMark (10). Functional annotation was based on BLASTp results with the NR databases and the Kyoto Encyclopedia of Genes and Genomes (KEGG). tRNA genes were directly predicted with tRNAscan-SE (11). The draft genome sequence consists of one rRNA operon, 46 tRNA genes, and 5,112 CDSs with an average length of 942 bp. Among the CDS products, 3,720 proteins were assigned to Clusters of Orthologous Groups (COG) families (12) and 3,952 proteins were assigned biological functions. A total of 2,235 proteins of *R. solanacearum* SD54 had KEGG orthologs, and these proteins were involved in 177 pathways. In addition, 284 proteins have no known match to any proteins in the databases. In a comparison with the known proteins of the *Ralstonia* genus, 4,603 proteins of *R. solanacearum* SD54 have orthologs.

### Nucleotide sequence accession numbers.

The genome sequence of *R. solanacearum* strain SD54 has been deposited in the GenBank database under the accession no. ASQR00000000. The version described here is accession no. ASQR02000000.
